# Impact of the COVID-19 pandemic on audiology services in South Africa: A preliminary study

**DOI:** 10.4102/hsag.v28i0.2318

**Published:** 2023-08-29

**Authors:** Katerina Ehlert

**Affiliations:** 1 Department of Speech-Language Pathology and Audiology, School of Healthcare Sciences, Sefako Makgatho Health Sciences University, Pretoria, South Africa

**Keywords:** COVID-19, pandemic, audiological services, audiology, impact, knowledge, attitudes

## Abstract

**Background:**

Hygiene-, work practices, travel, personal interactions and access to healthcare services changed for the majority of the world during the pandemic.

**Aim:**

This study aimed to discover the knowledge, impact and attitudes towards COVID-19 on the professional practices of public and private sector audiologists in South Africa.

**Setting:**

The study included 76 audiologists registered with the Health Professions Council of South Africa (HPCSA) and employed in the public, educational, tertiary or private practice and private sector in South Africa.

**Methods:**

A cross-sectional self-administered electronic survey study design was implemented.

**Results:**

Audiologists had appropriate knowledge regarding COVID-19. During hard lockdown, 69% of respondents saw less than 40% of their usual patient load, only 31% saw 60% – 100% of their usual patient load. During lower lockdown levels, majority of respondents (73.7%) saw 60% – 100% of their patient load while 26.3% still saw less than 40% of their usual patient load. Only hearing aid reprogramming, hearing aid trouble shooting, cochlear implant pre-counselling and adult hearing screening could be offered via tele-audiology. The main challenges faced were fear of infection, infection control measures in the workplace, accessibility and limited services provided during the various lockdown levels.

**Conclusion:**

The pandemic and lockdown levels had a definite impact on audiological service provision and many adaptations regarding service delivery and infection control in the workplace were required.

**Contribution:**

The relevance of this work for health services is the identification of the challenges experienced by audiologists during the pandemic and the opportunities to prepare for the future.

## Introduction

The COVID-19 pandemic has had a profound impact on various aspects of life, affecting almost all people worldwide indiscriminately, albeit to varying degrees. It is evident that there will be lasting changes in behaviour and practices as a result of this global crisis (Blumenthal et al. 2020). Hygiene practices, travel, work practices, personal interactions and access to services have all been affected for the majority of the world’s population (Eikelboom et al. 2021; Manchaiah et al. 2022).

The healthcare industry has needed to reconsider the traditional face-to-face delivery of clinical services because of the pandemic’s person-to-person transmission of a respiratory illness that originated in December 2019 in China and quickly spread globally (Blumenthal et al. 2020). The pandemic surprised everyone with its sudden onset and rapid spread. Many people and industries, including healthcare, were unprepared for the consequences. In response, national lockdowns were introduced, instructing people to stay at home and follow social distancing guidelines to limit the spread of virus (Gunjawate et al. 2021; Parmar, Beukes & Rajasingam 2022). In March 2020, the South African government announced the first of a series of national lockdowns intended to slow the spread of the disease and ease the growing pressure on the already overburdened South African healthcare system (South African Government 2020).

Some of the effects of the pandemic and lockdown levels included:

- reduced access to healthcare with hospitals and clinics being overwhelmed with COVID-19 patients, routine medical care and elective procedures were delayed or cancelled, leading to reduced access to healthcare for many people (American Medical Association 2021),
- telehealth: telehealth, or remote healthcare services, became more prevalent during the pandemic as people sought to limit in-person contact. This had been particularly important for audiology services, which often involve close proximity between patients and healthcare providers (American Academy of Audiology [AAA] 2021),
- changes in patient behaviour: patients had been more hesitant to seek healthcare services during the pandemic, particularly in the early stages when there was a lot of uncertainty about the virus. This had led to delayed diagnosis and treatment for many health conditions, including hearing loss (Centers for Disease Control and Prevention [CDC] 2021),
- changes in audiology practices: audiologists had to adapt to new protocols to ensure the safety of their patients and staff. This included increased use of personal protective equipment (PPE), enhanced cleaning and disinfection procedures and changes in scheduling and patient flow to limit the number of people in the clinic at any given time (American Speech-Language-Hearing Association [ASHA] 2021),
- increased awareness of hearing loss: the pandemic has highlighted the importance of communication and the challenges faced by people with hearing loss, particularly with the use of masks and social distancing.

This had led to increased awareness of hearing loss and the need for audiology services (The Hearing Review 2021).

Throughout the first, second, third and fourth waves of the COVID-19 pandemic, lockdown measures were implemented, resulting in significant disruptions to healthcare services, including nonessential services. Audiology departments had to cancel many existing clinical appointments, and only urgent cases were attended to, with audiologist-patient contact minimised. Moreover, many audiology professionals and practices lacked the necessary skills or equipment to provide audiology services remotely via telehealth, despite telehealth being approved as an acceptable service delivery method by the Health Professions Council of South Africa (HPCSA) (Ballachanda et al. 2020; Gunjawate et al. 2021; Parmar et al. 2022; HPCSA 2022).

The COVID-19 pandemic has significantly impacted healthcare and audiology services. However, providers have adapted to the new challenges and continued to provide crucial care to their patients. No studies have been conducted regarding the effects of the pandemic on audiologists in South Africa. This study aims to investigate the knowledge, impact and attitudes of public and private sector audiologists towards COVID-19 and its impact on their professional practices in South Africa. The findings of this study will inform professional bodies and employers about the impact of COVID-19 on audiology, enabling them to provide the necessary support and plan for the future of audiology services, including telehealth. Understanding how audiology services were affected during the pandemic and how audiologists responded to the changing needs of patients is essential in adapting and responding to these changes. Such perspectives can guide the necessary short- and medium-term changes for audiology in South Africa.

## Methods

### Study design

This study involved a cross-sectional self-administered electronic survey design to determine the impact of COVID-19 on professional practices of audiologists in South Africa. The main aim of the study was to determine the impact (including knowledge, practices and attitudes) of COVID-19 on professional practices of private and public audiologists in South Africa.

### Data collection sites, population and sampling

The population for the study was all hearing care professionals (audiologists, acousticians and audiometrists) registered with the HPCSA and employed in the public, educational, tertiary or private sector in South Africa, as well as hearing care professionals who work in private practice. Simple random sampling was applied, with a target population size of 344 for this study. The total number of hearing care professionals registered with HPCSA was 3266. A 95% confidence interval with a 5% margin of error was used to calculate the target population size.

### Data collection tool

The questionnaire used in the study was adapted from surveys performed in India and the United Kingdom (Gunjawate et al. 2021; Saunders & Roughly 2020) and validated for the South African context through a pilot study. Questions were removed, added and rephrased in order to improve the questionnaire. Google forms were used to develop and distribute the questionnaire electronically. The questionnaire included demographic information, practices during the pandemic, attitudes of audiologists and knowledge of COVID-19. Open and closed-ended questions were included, and the knowledge of COVID-19 was determined using multiple choice and true or false format questions.

### Data collection procedure

Following ethical clearance, data collection was done through online distribution of the questionnaire link via social media, email lists and other online platforms including professional bodies such as HPCSA, South African Speech-Language and Hearing association (SASLHA) and South African Association of Audiologists (SAAA). Informed consent and clear instructions for completing the questionnaire were included in the electronic questionnaire links. As respondents completed the questionnaire, the data were automatically collected and stored in Google Forms. The researcher downloaded the data for analysis.

### Data analysis

The first step involved data cleaning. This included removing any incomplete or irrelevant responses from the dataset. For example, if a respondent did not answer a particular question, or if they provided an answer that was clearly unrelated to the question, those responses were excluded from the analysis. Quantitative analysis of closed-ended questions was scrutinised using statistical methods such as descriptive statistics (e.g. mean, frequencies, percentages) and inferential statistics (Wilcoxon signed-rank test) was used to determine whether there was a statistical difference (*p* < 0.05) between services during hard- and lower lockdown levels and to identify patterns, trends and relationships among the variables. The variables considered in this study were knowledge on COVID-19 analysed using a memorandum, practices during the pandemic analysed using inferential statistics and attitudes of audiologists using descriptive statistics. Analysis of open-ended questions included thematic analysis, which involved coding and categorising the responses into themes or patterns to identify key issues, concerns or perspectives that emerge from the data.

### Ethical considerations

Ethical clearance to conduct this study was obtained from the Sefako Makgatho Health Sciences University Research Ethics Committee (No. SMUREC/H/266/2021:UG), prior to the onset of data collection. Electronic informed consent was obtained from all respondents before completing the questionnaire.

## Results

A total of 76 audiologists in South Africa participated in the study. A response rate of 23% was obtained, which is just below the recommended 25% or more for surveys (Genroe 2019). Table 1 describes the respondent demographics.

**TABLE 1:** Respondents demographic information (*N* = 76).

Demographic information	Category	*n*	%
Gender	Female	19	25.0
	Male	10	13.2
	No response	47	61.8
Qualification	Bachelor’s degree	53	69.7
	Master’s degree	16	21.1
	Doctorate (PhD)	4	5.3
	Doctorate of Audiology	3	3.9
Occupation	Audiologist	76	100.0
	Audiometrist	0	0.0
	Acoustician	0	0.0
Work setting	Public	39	51.3
	Private	30	39.5
	Tertiary	5	6.6
	Educational	1	1.3
	Other†	1	1.3
Province	Gauteng	34	44.7
	Limpopo	5	19.7
	Mpumalanga	8	10.5
	Western Cape	8	10.5
	North West	6	7.9
	KwaZulu-Natal	3	3.9
	Northern Cape	1	1.3
	Free State	1	1.3
	Eastern Cape	0	0.0

† , Special Severe Intellectual Disabilities (SID) resource centre.

### Knowledge of COVID-19

General questions about COVID-19 and management of COVID-19 patients were included. Respondents were required to respond with true or false. The majority of the respondents (93.7%, *n* = 71) were able to correctly identify the core symptoms of COVID-19, the way it can spread from person to person (97.4%, *n* = 74) and the social distancing that should be maintained from a person with COVID-19 (93.4%, *n* = 71).

### Audiology practices implemented during the COVID-19 pandemic

During the COVID-19 pandemic, various levels of restrictions were employed. Hard lockdown included level 5 restrictions that indicated a high COVID-19 spread with a low health system readiness. Level 1, 2, 3 and adjusted level 4 restrictions indicated a low or moderate COVID-19 spread with a low-to-moderate health system readiness (COVID-19 regulations by South African Government 2020). Hard lockdown was when operations had to be ceased and every person was expected to be at home as movements were restricted, which only allowed essential services to be provided. Lower lockdown levels were when restrictions were eased which permitted more services to be provided. The various lockdown levels had fluctuating effects on the practices of audiologists. Majority of the respondents (77%, *n* = 58) reported that hard lockdown had a major impact on the practices of audiologists while 20% (*n* = 15) reported some impact, 1% (*n* = 1) reported minimal impact and 2% (*n* = 2) reported no impact. During lower lockdown levels, 17% (*n* = 13) reported major impact, 42% (*n* = 32) reported some impact, 3% (*n* = 2) reported minimal impact and 10% (*n* = 8) reported no impact on audiological practices.

All respondents reported that their services had to adapt to the rules and regulations from the Department of Health during all lockdown levels. Patients had to be provided with prescreening case history forms (57%, *n* = 43), PPE had to be worn (88%, *n* = 67), social distancing was maintained in the workplace (91%, *n* = 69) as well as hands and surroundings had to be constantly sanitised or disinfected (97%, *n* = 74). Table 2 summarises the precautionary measures that had to be put in place at the workplace.

**TABLE 2:** Precautionary measures that had to be put in place at the workplace (*N* = 76).

Precautionary measures in the workplace	*n*	%
Hand disinfectant was available at various places in the clinic	74	97.4
Waiting area was arranged for social distancing	69	90.8
Masks were provided at the clinic for patients	24	31.6
Patients and audiology staff had to wear masks	67	88.2
Temperature measurement at facility upon arrival	60	78.9
COVID-19–related history was asked at the appointments	43	56.6
COVID-19 screening questionnaire was taken upon arrival	54	71.1
PCR testing was requested before in-person visit	10	13.2
Other: PPE such as screens, gloves and aprons was used at home visits and old age homes	5	6.6

PPE, personal protective equipment; PCR, polymerase chain reaction

Appointments of patients were often postponed, and less patients were seen per day in order to accommodate these adaptations. During lower lockdown levels, the patient loads could increase, but still not to pre-pandemic levels. During hard lockdown, 69% (*n* = 52) of respondents saw less than 40% of their usual patient load; only 31% (*n* = 24) saw 60% – 100% of their usual patient load. During lower lockdown levels, majority of respondents (73.7%, *n* = 56) saw 60%–100% of their patient load while 26.3% (*n* = 20) still saw less than 40% of their usual patient load. Table 3 summarises the main aspects that were impacted by the pandemic and the way audiologists practiced audiology during the various lockdown levels.

**TABLE 3:** Effects of pandemic on audiological practices (*N* = 76).

Lockdown level	All services stopped		Some services deferred		Seeing less patients per day		Less patients referred to me		Could not do home visits		Could not do home visits to old age homes		Had to start using PPE	
	*n*	%	*n*	%	*n*	%	*n*	%	*n*	%	*n*	%	*n*	%
Hard lockdown	11	14.5	34	44.7	55	72.4	40	52.6	16	21.1	18	23.7	11	14.5
Lower lockdown	2	2.8	16	22.5	42	59.2	26	36.6	10	14.1	13	18.3	2	2.8

PPE, personal protective equipment.

The main challenges faced by patients were hesitancy to come to the clinic because of infection risk (84%, *n* = 64) and difficulties accessing the clinic because of public restrictions (47%, *n* = 36). On the other hand, the main challenges faced by audiologists were patients unable to attend appointments (88%, *n* = 67), high infection risk for patients and employees (65%, *n* = 49) and infection control measures in the workplace (46%, *n* = 35). All the challenges faced by patients and audiologists during the pandemic are listed in Table 4.

**TABLE 4:** Challenges faced by patients and audiologists during the pandemic (*N* = 76).

Challenges faced	*n*	%
**Challenges faced by patients as reported by audiologists**		
Patients are hesitant to come to the clinic because of possible infection risk	64	84.2
Services are not offered at most or all practices	17	22.4
It is difficult to access the clinic because of public restrictions	36	47.4
There is lack of clear direction regarding when and how services will resume	14	18.4
Patients are not able to access teleservices	3	3.9
Patients are reluctant to access teleservices	2	2.6
Other: ‘patients would say they are in isolation or ill’, ‘the hospital has their own protocol’, ‘people get irritated with lack of funds’, ‘it was difficult to follow-up due to intervention disconnection and missing appointments’, ‘patients not coming to appointments due to having COVID-19’ ‘some patients could not come for appointments as they did not have money for transport because they were retrenched at work during the pandemic’.	7 - - - - - -	9.2 - - - - - -
**Challenges faced by audiologists in the workplace**		
High infection risk for employees and patients	49	64.5
Hygiene/infection control measures in workplace	35	46.1
Not able to provide full services needed by patients	29	38.2
Less employees available to provide services	18	23.7
Patients not being able to come in for appointments	67	88.2
Other	1	1.3

The Wilcoxon signed-rank test was used to determine whether there is a statistical difference (*p* < 0.05) between services during hard- and lower lockdown levels and between adults and children. Results indicated that there was no statistical significance between audiological services offered during hard and lower lockdown levels. Urgent audiological cases were addressed during all lockdown levels, and in general most cases were not postponed for more than 4 weeks. Table 5 describes how audiological services were affected by hard and lower lockdown levels for adults and children.

**TABLE 5:** Audiological services affected by hard- and lower lockdown levels (*N* = 76).

Audiological services	Urgent cases performed in less than 72 h		Case was postponed for no longer than 4 weeks		Case was postponed for no longer than 12 weeks		Non-urgent cases were postponed for longer than 12 weeks		Do not provide this service		Could not provide this service	
	*n*	%	*n*	%	*n*	%	*n*	%	*n*	%	*n*	%
**Audiometry assessment**												
**Adult**												
Hard lockdown levels	50	65.8	14	18.4	3	3.9	5	6.6	1	1.3	3	3.9
Lower lockdown levels	50	65.8	15	19.7	1	1.3	1	1.3	1	1.3	2	2.6
**Children**												
Hard lockdown levels	51	67.1	15	19.7	1	1.3	3	3.9	3	3.9	3	3.9
Lower lockdown levels	50	65.8	17	22.4	0	0	0	0	2	2.6	1	1.3
**Hearing screening**												
**Adult**												
Hard lockdown levels	0	0	12	15.8	4	5.2	2	2.6	9	11.8	9	11.8
Lower lockdown levels	0	0	11	14.5	2	2.6	1	1.3	6	7.9	3	3.9
**Children**												
Hard lockdown levels	48	63.2	15	19.7	0	0	2	2.6	7	9.2	4	5.2
Lower lockdown levels	50	65.8	13	17.1	0	0	0	0	60	78.9	1	1.3
**Electrophysiological measures**												
**Adult**												
Hard lockdown levels	27	35.5	16	21.1	1	1.3	3	3.9	20	26.3	8	10.5
Lower lockdown levels	29	38.2	17	22.4	2	2.6	2	2.6	15	19.7	5	6.6
**Children**												
Hard lockdown levels	30	39.5	16	21.1	4	5.2	0	0	18	23.7	7	9.2
Lower lockdown levels	28	36.8	21	27.6	2	2.6	0	0	15	19.7	4	5.3
**Supply and fitting of hearing aids**												
**Adult**												
Hard lockdown levels	33	43.4	26	34.2	4	5.3	6	7.9	3	3.9	4	5.3
Lower lockdown levels	39	51.3	22	28.9	3	3.9	1	1.3	5	6.6	0	0
**Children**												
Hard lockdown levels	41	53.9	15	19.7	22	28.9	3	3.9	6	7.9	2	2.6
Lower lockdown levels	42	59.2	22	28.9	1	1.3	1	1.3	4	5.3	0	0
**Hearing aids troubleshooting**												
**Adult**												
Hard lockdown levels	42	55.3	17	22.4	5	6.6	5	6.6	5	6.6	2	2.6
Lower lockdown levels	39	51.3	22	28.9	4	5.3	1	1.3	4	5.3	0	0
**Children**												
Hard lockdown levels	45	59.2	18	23.7	3	1.3	3	1.3	3	1.3	2	2.6
Lower lockdown levels	38	50	23	30.3	4	5.3	0	0	5	6.6	0	0
**Cochlear implant switch on**												
**Adult**												
Hard lockdown levels	15	19.7	8	10.5	2	2.6	0	0	38	50	12	15.8
Lower lockdown levels	13	17.1	9	11.8	2	2.6	2	2.6	36	47.4	8	10.5
**Children**												
Hard lockdown levels	14	18.4	11	14.5	0	0	3	1.3	37	48.7	10	13.2
Lower lockdown levels	14	18.4	13	17.1	2	2.6	0	0	35	46.1	6	7.9
**Cochlear implant fitting**												
**Adult**												
Hard lockdown levels	13	17.1	9	11.8	0	0	1	1.3	37	48.7	14	18.4
Lower lockdown levels	12	15.8	9	11.8	3	3.9	1	1.3	36	47.4	9	11.8
**Children**												
Hard lockdown levels	14	18.4	9	11.8	3	3.9	3	3.9	36	47.4	10	13.2
Lower lockdown levels	14	18.4	11	14.5	3	3.9	1	1.3	35	46.1	6	7.9
**Cochlear implant troubleshooting**												
**Adult**												
Hard lockdown levels	11	4.5	9	11.8	2	2.6	1	1.3	38	50	14	18.4
Lower lockdown levels	12	15.8	9	11.8	2	2.6	2	2.6	35	46.1	10	13.2
**Children**												
Hard lockdown levels	11	14.5	12	15.8	10	13.2	3	3.9	36	47.4	12	15.8
Lower lockdown levels	14	18.4	12	15.8	2	2.6	1	1.3	35	46.1	6	7.9
**Vestibular assessment**												
**Adult**												
Hard lockdown levels	23	30.3	12	15.8	2	2.6	4	5.3	18	23.7	16	21.1
Lower lockdown levels	21	27.6	13	17.1	6	7.9	6	7.9	16	21.1	8	10.5
**Children**												
Hard lockdown levels	21	21.6	15	19.7	4	5.3	6	7.9	8	23.7	11	14.5
Lower lockdown levels	26	34.2	12	15.8	4	5.3	4	5.3	35	46.1	35	46.1
**Vestibular rehabilitation**												
**Adult**												
Hard lockdown levels	18	23.7	15	19.7	4	5.3	4	5.3	19	25.0	15	19.7
Lower lockdown levels	23	30.3	11	14.5	7	9.2	6	7.9	14	18.4	9	11.8
**Children**												
Hard lockdown levels	19	25.0	15	19.7	5	6.6	7	9.2	18	23.7	11	14.5
Lower lockdown levels	22	28.9	14	18.4	7	9.2	3	3.9	16	21.1	8	10.5

The main services that could not be provided via tele-audiology included newborn hearing screening (86%, *n* = 65), general audiological testing (75%, *n* = 57), hearing aid fitting and aural rehabilitation in adults (74%, *n* = 56), repair of hearing aids (51%, *n* = 39) and hearing aid follow-up and reprogramming (50%, *n* = 37). Table 6 describes the audiological services that could and could not be provided via tele-audiology for both adults and children.

**TABLE 6:** Tele-audiology service provision for adults and children (*N* = 76).

Audiological services	Services that were provided via tele-audiology		Services could not be provided via tele-audiology	
	*n*	%	*n*	%
Newborn hearing screening	10	13.5	65	85.5
General audiological testing	14	18.9	57	75.0
Pre-cochlear implant counselling	27	36.5	14	18.4
Cochlear implant switch on	7	9.5	26	34.2
Cochlear implant follow-up programming or fitting	13	17.6	20	26.3
Troubleshooting of hearing aids	34	45.9	21	27.6
Repair of hearing aids	15	20.3	39	51.3
Troubleshooting repair of cochlear implant	8	10.8	20	26.3
Hearing aid fitting and aural rehabilitation (adults)	13	17.6	56	73.7
Vestibular testing and vestibular rehabilitation	14	18.9	31	40.8
Hearing aid follow-up and reprogramming	37	50.0	37	50.0
Adult hearing screening	19	25.7	19	25.7

### Attitudes towards the COVID-19 pandemic

A Likert scale was used to measure the attitudes of audiologists towards the COVID-19 pandemic. Five statements were included, and respondents were asked to indicate whether they strongly agree, agree, neutral or disagree. The majority of respondents (> 50%) continued to provide services during the pandemic and did not cancel appointments because of COVID-19 symptoms in patients. The respondents’ responses are described in Table 7.

**TABLE 7:** Attitudes towards the COVID-19 pandemic (*N* = 76).

Statement	Strongly agree		Agree		Neutral		Disagree		Strongly disagree	
	*n*	%	*n*	%	*n*	%	*n*	%	*n*	%
Would you continue providing services even if there is a positive case in the workplace?	39	51.3	16	21.1	9	11.8	6	7.9	6	7.9
Would you provide treatment to patients exhibiting symptoms of COVID-19?	24	31.6	30	39.5	8	10.5	6	7.9	8	10.5
Are you scared of contracting COVID-19 from coming in contact with people in the workplace?	7	9.2	7	9.2	35	46.1	9	11.8	18	23.7
Did you not permit patients for assessment/therapy if they complained of any symptoms related to COVID-19?	8	10.5	4	5.3	12	15.8	25	32.9	27	35.5
COVID-19 was successfully controlled in the workplace.	4	5.3	6	7.9	19	25.0	30	40.0	17	22.4

## Discussion

The study conducted sheds light on the impact of the COVID-19 pandemic on audiology services in South Africa. Of the total participants, 40% worked in private practice, and 50% worked in public health. The study included participants from all the provinces in South Africa except for the Eastern Cape. The findings of the study suggest that audiologists had sufficient knowledge about COVID-19, and appropriate infection control measures were implemented. This is in contrast to a study conducted in India, which reported that audiologists had poor practices towards infection control measures, especially hand washing, highlighting the need for better awareness among audiologists about appropriate and standard infection control measures (Gunjawate et al. 2021). It can be inferred that the consistent media attention, training provided at the workplace and COVID-19 regulations by the South African Government (2020), Department of Health websites (NDOH 2022) and the CDC (2022) ensured that healthcare workers had access to the required information regarding the virus’s knowledge, transmission and precautionary measures.

The study also reported that the hard lockdown significantly impacted audiological services, as 77% of respondents experienced a decrease in their patient load. Even during lower lockdown levels, the majority of respondents (74%) saw 60% – 100% of their patient load, while 26% still saw less than 40% of their usual patient load. Consequently, many patients had to delay or cancel appointments, resulting in reduced access to services. Patients were hesitant to come to the clinic because of the risk of infection. Tele-audiology became mandatory, and audiologists had to adapt quickly to provide these services. A study conducted in South Africa by Swanepoel et al. (2020) supports the finding that lockdown levels significantly affected audiological services. The study found that during hard lockdown, 69% of respondents saw less than 40% of their usual patient load and only 31% saw 60% – 100% of their usual patient load. Moreover, appointments were often postponed, and fewer patients were seen per day (72%) to comply with the restrictions. Another study conducted in the United Kingdom by Atkinson, Shipway and Woodcock (2021) reported that audiology services were reduced by an average of 62% across the country during the first wave of the pandemic, with some services being closed completely. During the second wave of the pandemic, which occurred during lower lockdown levels, services were still reduced by an average of 38%.

The services that could not be provided via tele-audiology in the current study included newborn hearing screening, general audiological assessment, hearing aid fitting and aural rehabilitation for adults, repair of hearing aids, vestibular assessment and rehabilitation and cochlear implant switch on. The services that could be provided via tele-audiology included troubleshooting of hearing aid, hearing aid follow-up and reprogramming, pre-cochlear implant counselling and adult hearing screening. Similar studies conducted in the United Kingdom and internationally reported tele-audiology barriers for verification, paediatrics, earmould adjustments, diagnostics, complex patients and assessing mental health. The tele-audiology service delivery difficulties included suitable technology and technical support, data protection, competence and confidence of audiologists, rapport and patient interaction and change in working patterns. Accessibility challenges for patients were sight difficulties, severe hearing loss and no access to technology (Eikelboom et al. 2021; Manchaiah et al. 2022; Parmer et al. 2022). Similarly, the current study highlighted the challenges and limitations in tele-audiology service delivery, emphasising the need for audiologists to consider improvement and development in the capacity for telehealth service provision.

In the present study, urgent cases were treated similarly during hard and lower lockdowns for both adults and children. The most common services provided were audiological assessments, hearing aid fittings and troubleshooting, followed by electrophysiological measures and vestibular assessments and rehabilitation. These findings are supported by AAA (2020), which reported that emergency audiological services were still available during the pandemic. For example, if a patient experienced sudden hearing loss or had an issue with their hearing aid that required immediate attention, they could still seek emergency services from their audiologist. Furthermore, amplification in children is considered an emergency, as the impact of hearing loss can cause lifelong delays (Yoshinaga-Itano 2020).

The main challenges faced by audiologists in this study were patients not being able to attend appointments, a high infection risk for employees and patients, hygiene and infection control measures in the workplace, an inability to provide the full range of services required and fewer employees being available to provide services. Patients reported hesitance to come to the clinic because of the possible risk of infection, difficulty accessing the clinic and not all services being offered. These outcomes are supported by AAA (2020) and ASHA (2020), which identified limited availability of PPE, reduced access to audiology equipment, reduced patient volumes, limited tele-audiology services and compliance with infection control measures as challenges faced by audiologists.

Audiologists’ attitudes towards the COVID-19 pandemic indicated that the majority (72%) would continue providing services even if there was a positive case in the workplace, and they would provide treatment to patients exhibiting symptoms of COVID-19. Several studies support the finding that audiologists are committed to providing services during the pandemic, despite the risks. A scoping review found that 71% of respondents reported that they continued to provide in-person services during the pandemic. The review also found that audiologists were implementing a range of measures to minimise the risk of COVID-19 transmission, including wearing PPE, conducting screenings of patients prior to appointments and increasing cleaning and disinfecting protocols in their clinics (Aggarwal et al. 2021). Another study surveyed audiologists in the United Kingdom and found that 75% of respondents continued to provide in-person services during the pandemic, with 64% of those reporting that they had modified their practices in response to the pandemic (Saunders & Roughley 2021). This highlights the commitment of audiologists to continuing service delivery during the pandemic, despite the risks.

The findings of this study should be interpreted with caution, as the sample size was relatively small. However, the study represents audiologists from eight of the nine provinces in South Africa. Despite these limitations, the study provides important insights into the impact of COVID-19 on audiology services in South Africa. The study highlights the importance of infection control measures, tele-audiology and adapting to new technologies to ensure that patients receive the care they need, even during a pandemic. Overall, the findings of this study are consistent with other studies that report the impact of COVID-19 on audiology services worldwide.

## Conclusion

The pandemic and associated lockdowns had a significant impact on the delivery of audiological services, requiring adaptations in service provision and infection control measures in the workplace. Tele-audiology was implemented to a limited extent because of equipment, access and connectivity challenges. This work is relevant for health services as it identifies the challenges experienced by audiologists during the pandemic and opportunities to prepare for the future. The COVID-19 pandemic had a profound sociological, psychological and professional impact. Although the pandemic has passed with lifestyle changes and effective treatments and vaccines, this study highlights the need for the audiology profession to adopt new clinical technologies and business models for improved reimbursement, service delivery and patient-centred care. COVID-19 has accelerated the future of healthcare, and audiologists have a unique opportunity to prepare for tele-audiology and improved service delivery to make it accessible for all.
